# Validation of the Italian Version of the SARC-F Questionnaire to Assess Sarcopenia in Older Adults

**DOI:** 10.3390/nu14122533

**Published:** 2022-06-18

**Authors:** Simone Perna, Clara Gasparri, Cinzia Ferraris, Gaetan Claude Barrile, Alessandro Cavioni, Francesca Mansueto, Zaira Patelli, Gabriella Peroni, Alice Tartara, Marco Zese, Mariangela Rondanelli

**Affiliations:** 1 Department of Biology, College of Science, University of Bahrain, Sakhir Campus, Zallaq P.O. Box 32038, Bahrain; 2 Endocrinology and Nutrition Unit, Azienda di Servizi alla Persona ‘‘Istituto Santa Margherita’’, University of Pavia, 27100 Pavia, Italy; clara.gasparri01@universitadipavia.it (C.G.); gaetanclaude.barrile01@universitadipavia.it (G.C.B.); alessandro.cavioni01@universitadipavia.it (A.C.); francesca.mansueto01@universitadipavia.it (F.M.); zaira.patelli01@universitadipavia.it (Z.P.); gabriella.peroni01@universitadipavia.it (G.P.); alice.tartara01@universitadipavia.it (A.T.); marco.zese01@universitadipavia.it (M.Z.); 3 Laboratory of Food Education and Sport Nutrition, Department of Public Health, Experimental and Forensic Medicine, University of Pavia, 27100 Pavia, Italy; cinzia.ferraris@unipv.it; 4 IRCCS Mondino Foundation, 27100 Pavia, Italy; mariangela.rondanelli@unipv.it; 5 Unit of Human and Clinical Nutrition, Department of Public Health, Experimental and Forensic Medicine, University of Pavia, 27100 Pavia, Italy

**Keywords:** sarcopenia, SARC-F, Italian, muscle, elderly, frailty

## Abstract

Background: SARC-F is a simple sarcopenia screening tool. This study aimed to examine the validity of the Italian version of SARC-F. Methods: A total of 97 elderly individuals (37/60 males/females, 65 years and older) who met the study’s selection criteria were included. SARC-F was translated into the Italian language in a culturally responsive manner. The total score was calculated by adding the scores on the five items. The participants were divided into two groups according to the total score (SARC-F < 4 vs. SARC-F ≥ 4), and their associations with various factors (handgrip test, chair stand test, and Skeletal Muscle Index assessed by DXA) have been examined by gender. In addition, the tool’s validity was analyzed by comparing it with different international working group diagnostic criteria for sarcopenia. Results: The total prevalence of sarcopenia according to the SARC-F was 14.2% and, specifically, 12.8% among men and 14.3% in women. The sensitivity of the SARC-F was (male (M): 11–50% and female (F): 22–36%) medium-low compared with the European, international, and Asian criteria of sarcopenia; however, SARC-F showed a high specificity (M: 77.3–100% and F: 79.5–100%) and a moderate Cronbach’s alpha coefficient of (0.669 (CI95%: 0.358–0.830). The participants in the SARC-F ≥ 4 group had poorer handgrip for EWGSOP2 (*p* < 0.001) and chair stand (*p* < 0.001) than the participants in the SARC-F < 4 group. Conclusions: The Italian language version of SARC-F showed high specificity, moderate reliability, and good associations with other predictive tests. The Italian version of SARC-F appears to be a useful screening tool for the diagnosis of sarcopenia in Italian elderly populations.

## 1. Introduction

Sarcopenia is a progressive syndrome that affects skeletal muscle mass and strength; it occurs more frequently in the elderly and is associated with an increased risk of adverse outcomes including falls, fractures, physical disability, and mortality [1]. Sarcopenia has been recognized as a disease entity with the assignment of the International Classification of Diseases, Tenth Revision, Clinical Modification (ICD-10-CM) (M62.84) code in September 2016 [2]. The diagnosis of sarcopenia is based on different tools for the measurement of muscle mass or strength and physical performance. For this reason, the prevalence estimations of sarcopenia are variable, reflecting the different approaches to its definition and the differences across various populations [3].

The most recent update regarding sarcopenia was carried out by the European Working Group on Sarcopenia in Older People 2 (EWGSOP2) in 2018 [1]. SARC-F is a simple and valid tool used in the screening stages for assessing sarcopenia risk, and it is developed by Malmstrom and Morley [4].

Introducing the SARC-F questionnaire into clinical practice is recommended by EWGSOP2, due to its accuracy in screening for sarcopenia and the fact that it is an inexpensive and convenient method for sarcopenia risk screening, making it easily used in community healthcare and other clinical settings where DXA or other imaging techniques are not available [1].

SARC-F is a five-item self-reported questionnaire with items about strength, walking ability, rising from a chair, stair climbing, and experiences with falls [4]; SARC-F has a low-to-moderate sensitivity and a very high specificity in predicting low muscle strength, meaning that SARC-F will mostly detect severe cases [5].

To date, SARC-F has been translated and validated in several languages, from the original English language to Spanish [6], French [7], Portuguese [8], Korean [9], and Chinese [10]. However, an Italian version has not been validated yet.

A recent study by Yu et al. summarized the main strengths/advantages and limitations/disadvantages of current sarcopenia screening and diagnostic tools, reporting that, relative to the EWGSOP algorithm, no validation studies have evaluated this tool’s sensitivity and specificity. The PPV and NPV of this tool are unknown and there is limited clinical utility in screening older adults for sarcopenia due to the high proportion of subjects selected to further undergo muscle assessment [11].

In addition, anthropometric prediction equations indicated a good discriminatory tool as a “rule-out” screening test, but they are not yet validated in care facility residents or hospital inpatients and not yet validated in the non-Caucasian population. The major limitation of SARC-F, as reported for the first time by Malmstrom, is that its low sensitivity may miss people who are sarcopenic but classified as “not sarcopenic” according to SARC-F [12].

Given this background, the purpose of the present study is, therefore, to translate and validate SARC-F into Italian. The translation process has been divided into two consecutive parts: (1) the translation of the questionnaire from English to Italian and its language validation and (2) the clinical validation of the Italian SARC-F to assess the performance of the SARC-F questionnaire in a cohort of elderly Italian subjects, according to the various existing definitions of sarcopenia tools.

## 2. Materials and Methods

### 2.1. Population and Study Design

The present study’s patient pool included subjects aged over 65 years, recruited from 2020 to 2022 at the Dietetic and Metabolic Unit of the “Santa Margherita” Institute, University of Pavia, Italy. Informed written consent was provided by all participants, and the research protocol was approved by the Ethics Committee of the University of Pavia (ethical code number: 2207/01022021).

This is a cross-sectional study reporting the following inclusion criteria: (1) admission to a geriatric care unit for functional loss secondary to a non-disabling medical disease, (2) aged 65 years or older, and (3) willingness to participate and to provide signed informed consent. At the time of admission, the patients were not diagnosed with disabling diseases (such as neurological diseases, hip fractures, or amputations).

Exclusion criteria included the following: subjects affected by acute illness, severe liver disease, or kidney dysfunction (acute kidney ‘risk, injury, failure’ or severe dementia (MMSE < 18 points).

### 2.2. The Questionnaire

The SARC-F questionnaire includes questions and their score related to 5 items:

(A) Strength: ‘‘How much difficulty do you have in lifting and carrying 10 pounds’’ (none = 0; some = 1; a lot or unable = 2);
(B) Assistance in walking: ‘‘How much difficulty do you have walking across a room’’ (none = 0; some = 1; a lot, use aids, or unable = 2);
(C) Rise from a chair: ‘‘How much difficulty do you have transferring from a chair or bed’’ (none = 0; some = 1; a lot or unable without help = 2);
(D) Climb stairs: ‘‘How much difficulty do you have climbing a flight of ten stairs’’ (none = 0; some = 1; a lot or unable = 2);
(E) Falls: ‘‘How many times have you fallen in the past year’’ (none = 0; 1/3 falls = 1; 4 falls or more = 2). The scale score ranges from the following Likert scale from 0 to 10 (0 = best to 10 = worst); patients who have a total score ≥ 4 are considered at risk of having sarcopenia [3].

### 2.3. Procedure

Based on a previous study [6], the translation process has been divided into two consecutive phases: (1) the translation of the questionnaire from English to Italian and cross-cultural validation of this translation and (2) the clinical validation of the Italian SARC-F to assess the performance of the SARC-F questionnaire according to the various existing definitions of sarcopenia (Supplementary File S1).

#### 2.3.1. Italian Translation and Cross-Cultural Validation of the SARC-F

To validate the original SARC-F questionnaire, it was first translated into Italian by two operators, back-translated into English by a native English operator, and then retranslated to Italian by another two operators. The original scale was compared to the retranslated version by the expert’s panel, without finding significant differences. The final Italian version was administered to 20 subjects to ensure it was comprehensible.

#### 2.3.2. Clinical Validation of the Italian SARC-F

Once SARC-F had been translated into Italian, a clinical validation study was performed with this SARC-F version to assess its performance for the diagnosis of sarcopenia. By conducting a cross-sectional study, the final version of the Italian SARC-F was administered to the population of the present study to assess its sensitivity (Se), specificity (Sp) positive predictive value (PPV), negative predictive value (NPV), and accuracy of the SARC-F according to some operational definitions of sarcopenia.

### 2.4. Statistical Analysis

All subjects enrolled in this study have been divided into two cohorts based on their SARC-F scores (SARC-F < 4 and SARC-F > 4) and into subgroups based on gender.

The Kolmogorov–Smirnov test was used to test the null hypothesis that a set of data comes from a normal distribution.

Continuous variable results were expressed as mean and standard deviations, and categorical variables were expressed as numbers and percentages.

To analyze the frequency between the components, according to the various definitions of sarcopenia, and to examine the relationships between SARC-F scores and each diagnostic tool, we applied the Chi-square test. In addition, the ROC curve for specificity and Cronbach’s alpha for reliability have been assessed. A Bland–Altman analysis was also carried out to detect whether there was a systematic bias in the test–retest data. Reliability (internal consistency) was assessed by using SARC-F Cronbach alpha. Concordance between the first and second-raters, the test–retest reliability, and agreement between SARC-F and both EWGSOP criteria was determined with kappa (k) statistics. The cut-off points for reliability were set as follows: k < 0, no agreement; 0–0.20, poor agreement; 0.21–0.40, fair agreement; 0.41–0.60, moderate agreement; 0.61–0.80 substantial agreement; 0.81–1, almost perfect agreement.

The data software used to perform the analysis was SPSS vs. 28.0 (IBM Corporation, Chicago, IL, USA); *p* value < 0.05 was defined as the main level for testing the null hypothesis.

We estimated the sample size required to achieve 0.8 power to detect the difference between the ROC curves using the “sample size: comparison of ROC curves” function in MedCalc Statistical Software 15.2. The estimated sample size was 100 (including 15 individuals with sarcopenia and 85 participants without sarcopenia).

## 3. Results

This study included 100 patients (37 males and 60 females). In the total sample, three patients were excluded because of falls and consequent hip fractures (two patients) and acute neurological disease (stroke) in one patient. Table 1 displays the average characteristics of the participants who were grouped according to their SARC-F scores. Across SARC-F participants’ sexes, a notable difference was found in terms of individual diagnostic items, with higher SMI, ALM/BMI, handgrip (kg), chair test (s), SPPB tot (pt), and gait speed (m/s) in males and the SARC-F > 4 group. Table 2 shows the prevalence of sarcopenia determined by SARC-F in association with other diagnostic criteria such as EWGSOP, IWGS, AWGS, and FNIH, which varied according to gender. Table 3 shows that the sensitivity of the SARC-F was low compared with the European, international, and Asian criteria of sarcopenia (male (M): 11–50% and female (F): 22–36%). However, SARC-F showed a high specificity (M: 77.3–100% and F: 79.5–100%)

**Figure 1:**
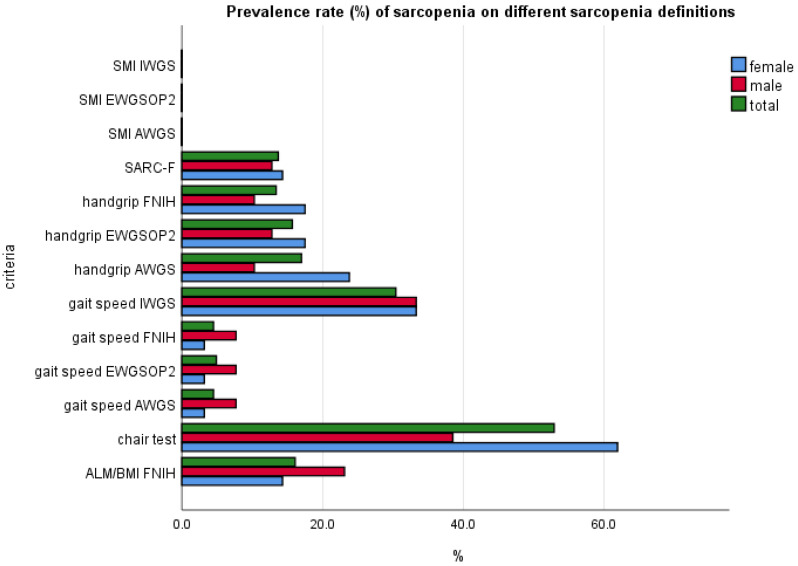
Prevalence rate (%) of sarcopenia on different sarcopenia definitions.

Figure 1 shows that the prevalence of sarcopenia according to SARC-F was 12.8% among men and 14.3% among women. The highest level of sarcopenia was recorded with gait speed IWOS criteria and for the chair test (over 60% in females). Handgrip by EWGSOP2 and gait speed AWOS had similar prevalence results with SARC-F.

**Figure 2:**
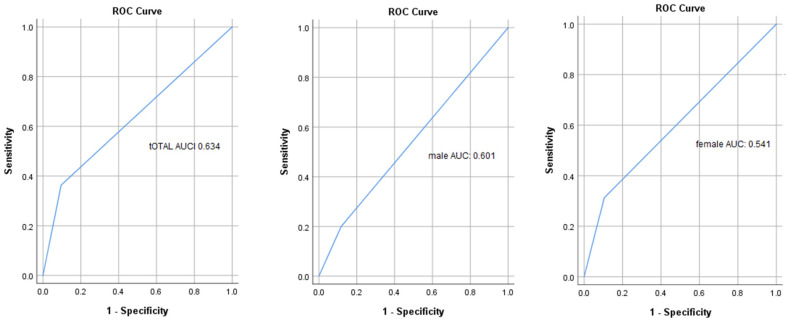
The receiver operator curve (ROC) of the SARC-F for sarcopenia based on European criteria. AUC, area under the curve; CI, confidence interval.

European criteria were examined with the receiver operator curve. The results indicated an area under the curve of 0.691 for men and 0.541 for women (Figure 2).

**Figure 3:**
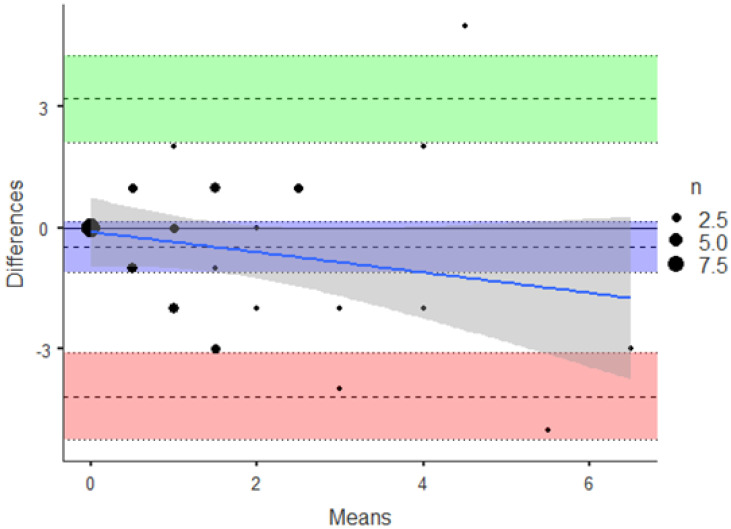
Bland–Altman and the Cronbach’s alpha test for reliability in a subgroup of subjects. The Bland–Altman test was performed (Figure 3). Internal consistency among the items was excellent with a Cronbach’s alpha with InterClass Correlation (CI 95%) at 0.669 (0.358–0.830) *p* < 0.001.

Figure 3 showed the internal consistency among the items that was excellent with a Cronbach’s alpha with InterClass Correlation (CI 95%) at 0.669 (0.358–0.830) *p* < 0.001.

## 4. Discussion

To our knowledge, this is the first study of the Italian population for verifying the validity and reliability of the Italian version of SARC-F.

Compared to the European, international, and Asian criteria of sarcopenia, the findings of this study showed that SARC-F showed a high specificity (M: 77.3–100%, F: 79.5–100%) and a moderate Cronbach’s alpha coefficient of (0.669 (CI95%:0.358–0.830) in line with the target results of the Korean SARC-F version. However, SARC-F showed a high specificity [9] and demonstrated a slightly better performance compared with the French and Spanish versions [7,13].

Therefore, the SARC-F Italian version appears to be suitable for ruling out and screening in older adults without sarcopenia.

Comparing the SARC-F < 4 group with the SARC-F > 4 group, the data showed lower performance and muscle strength as evidenced by the handgrip test and chair stand test.

In addition, patients with higher SARC-F > 4 showed lower SPPB and gait speed with consequences on the reduction in SMI and ALM. Furthermore, differences in the results of BMI have been recorded in the two groups of SARC-F.

These data provided evidence that SARC-F is a very simple and useful tool for screening sarcopenia, physical performance, as well as the health-related quality of life and frailty-related conditions.

The total prevalence rate of sarcopenia based on SARC-F was 14.3%, which was 12.8% among men and 14.3% in women, and this showed a clear gender difference compared with the prevalence rates obtained via other diagnostic criteria. Worth noting is that SARC-F appeared to be over screening females with sarcopenia, although similar data emerged in this population when using other diagnostic tools. We recorded that sarcopenia in the chair test was 61.9% in females and 38.5% in males; using handgrip EWGSOP2, sarcopenia in females was 17.5% and 12.8% in males. Within our sample when only applying the ALM/BMI FNIH criteria, we found higher sarcopenia in males at 23.1% versus 14.3% in females, while measuring the gait speed with FNIH criteria showed that sarcopenia in females was 3.2% and 7.7% in males.

This huge gap by gender has been reported in a recent study by Rolland et al. [14], which reported a prevalence rate of 16.7% among women. As reported in this study, the SARC-F response rate for each item invariably varied between gender and age.

SARC-F is a simple screening test with a high specificity and high negative predictive value, which makes it useful when ruling out the presence of sarcopenia in a clinical setting.

By using the cut-off limit of four points as the reference, we reported the lowest levels among men in both specificity and sensitivity, which were 77% and 11%, respectively. Similar data have been reported in the Polish version of SARC-F [15].

Given these biases in males, we recommend utilizing the DXA device to better detect sarcopenia in men due to the possibility of errors in detecting false positives with SARC-F.

Despite these limitations, the present study is the first to investigate the validity and application of SARC-F in the Italian version, applied in a cultural setting. Reliability involves good accuracy and precision. In our study, SARC-F accuracy was 0.67% higher than the Spanish version, which was 0.58% [13], and it was very similar to the Japanese version, which was 0.61% [16].

The Italian version of the SARC-F proved to be a good screening method that is easy to administer and minimally invasive for confident use in clinical practice. Reliability showed good general accuracy and precision.

The present study examined the utility of the Italian version of SARC-F for community-dwelling older adults. SARC-F was significantly related to measures of physical performance and muscle mass. Although the scale showed good construct validity, internal consistency was sufficient; furthermore, sensitivity was poor for detecting sarcopenia, whereas specificity was as good as in previous studies. The Italian version of SARC-F might be a useful screening tool for determining sarcopenia within clinical settings.

## 5. Conclusions

The Italian language version of SARC-F showed a high specificity, moderate reliability and good associations with other predictive tests. The Italian version of SARC-F appears to be a useful screening tool for the diagnosis of sarcopenia in Italian older adults.

## Figures and Tables

**Table 1:** Baseline Characteristics.

	M (*n* = 37)		F (*n* = 60)		Total (*n* = 97)	
	SARC-F < 4 (*n* = 33) Mean ± ds	SARC-F > 4 (*n* = 4) Mean ± ds	SARC-F < 4 (*n* = 52) Mean ± ds	SARC-F > 4 (*n* = 8) Mean ± ds	SARC-F < 4 (*n* = 85) Mean ± ds	SARC-F > 4 (*n* = 12) Mean ± ds
**BMI (kg/m^2^)**	27.71 ± 3.54	27.27 ± 3.11	28.31 ± 7.36	29.32 ± 6.98	28.08 ± 6.15	28.59 ± 5.83
**TM (kg)**	79.79 ± 10.25	75.64 ± 10.16	68.24 ± 16.89	71.39 ± 14.44	72.81 ± 15.63	73.02 ± 12.68
**FM (g)**	24,262.29 ± 7609.60	21,743.60 ± 5447.88	28,208.46 ± 12,858.59	30,655.12 ± 10658.77	26,648.35 ± 11,200.60	27,227.62 ± 9824.87
**FFM (g)**	52,682.21 ± 5338.61	51,239.80 ± 8036.05	38,087.75 ±	38,749.88 ± 6712.97	43,857.65 ± 8734.70	43,553.69 ± 9370.85
**BMC (g)**	2850.91 ± 410.25	2641.20 ± 490.95	1929.25 ± 4780.89	1978.25 ± 525.91	2293.63 ± 565.59	2233.23 ± 595.29
**FM %**	30.950 ± 7.14	29.760 ± 6.59	40.608 ± 9.09	43.175 ± 9.12	36.790 ± 9.59	38.015 ± 10.45
**Android %**	38.31 ± 10.98	32.22 ± 6.97	43.27 ± 13.54	46.06 ± 10.94	41.31 ± 12.76	40.74 ± 11.62
**Gynoid %**	30.84 ± 7.06	91.16 ± 127.61	43.84 ± 8.45	45.54 ± 9.41	38.70 ± 10.15	63.09 ± 77.55
**VAT (g)**	1662.18 ± 889.43	835.40 ± 313.25	1056.95 ± 719.66	1170.25 ± 452.27	1291.92 ± 838.96	1041.46 ± 425.18
**FFM arms (g)**	6329.62 ± 761.45	5459.60 ± 904.06	3826.77 ± 614.70	4072.00 ± 957.99	4816.27 ± 1402.46	4605.69 ± 1140.83
**Total arms (kg)**	9.12 ± 1.12	8.38 ± 1.10	6.99 ± 1.51	7.75 ± 957.99	7.83 ± 1.72	7.99 ± 1.33
**FFM legs (g)**	19,394.29 ± 2506.15	18,817.00 ± 4229.60	14,527.71 ± 2218.12	14,329.13 ± 2612.52	16,451.71 ± 3334.46	16,055.23 ± 3887.02
**Total legs (kg)**	27.52 ± 3.38	27.22 ± 4.75	25.82 ± 6.52	26.01 ± 5.44	26.49 ± 5.53	26.48 ± 5.02
**ASM (kg)**	25.72 ± 3.10	24.28 ± 5.09	18.35 ± 2.58	18.40 ± 3.28	21.27 ± 4.57	20.66 ± 4.87
**SMI**	8.88 ± 0.78	8.60 ± 1.04	7.62 ± 0.95	7.82 ± 1.01	8.12 ± 1.08	8.13 ± 1.05
**ALM/BMI**	0.94 ± 0.17	0.90 ± 0.21	0.67 ± 0.15	0.62 ± 0.16	0.78 ± 0.20	0.73 ± 0.23
**Handgrip (kg)**	37.32 ± 7.09	27.40 ± 5.94	20.81 ± 5.54	19.56 ± 9.02	27.19 ± 10.15	22.36 ± 8.72
**Chair test (s)**	14.27 ± 5.06	25.93 ± 6.93	15.92 ± 4.29	24.21 ± 6.61	15.28 ± 4.65	24.74 ± 6.47
**SPPB tot (pt)**	10.06 ± 1.84	5.80 ± 1.30	8.88 ± 1.91	6.00 ± 1.80	9.33 ± 1.96	5.93 ± 1.59
**gait speed (m/s)**	1.09 ± 0.30	1.95 ± 0.80	1.17 ± 0.29	2.03 ± 0.81	1.14 ± 0.29	2.00 ± 0.77

BMI, body mass index; Tm, total mass; FM, fat mass; FFM, fat free mass; BMC, bone mineral content; VAT, visceral adipose tissue, ASM, appendicular skeletal mass; SMI, skeletal muscle mass; ALM, appendicular lean mass, (SPPB) The short physical performance battery.

**Table 2:** SARC-F and Various Sarcopenia Definitions.

	M					F					Total				
	SARC-F < 4		SARC-F > 4		*p*	SARC-F < 4		SARC-F > 4		*p*	SARC-F < 4		SARC-F > 4		*p*
	*n*	%	*n*	%		*n*	%	*n*	%		*n*	%	*n*	%	
**Chair test**															
**No sarcopenia**	24	70.6	0	0	0.011	24	44.4	0	0	0.030	48	54.5	0	0	0.000
**Sarcopenia**	10	29.4	5	100	0.011	30	55.6	9	100	0.030	40	45.5	14	100	0.000
**European Working Group on Sarcopenia in Older People EWGSOP2**															
**No sarcopenia**	34	100	5	100		53	100	8	100		97	100	13	100	
**Sarcopenia**	0	0	0	0		0	0	0	0		0	0	0	0	
*SMI for EWGSOP2*															
**No sarcopenia**	34	100	5	100		53	100	9	100		87	100	14	100	
**Sarcopenia**	0	0	0	0		0	0	0	0		0	0	0	0	
*Handgrip for EWGSOP2*															
**No sarcopenia**	30	88.2	4	80	1.000	47	97	5	55.6	0.067	77	87.5	9	64.3	0.068
**Sarcopenia**	4	11.8	1	20	1.000	7	13	4	44.4	0.067	11	12.5	5	35.7	0.068
*Gait speed for EWGSOP2*															
**No sarcopenia**	31	91.2	5	100	1.000	52	96.3	9	100	1.000	83	94.3	14	100	0.804
**Sarcopenia**	3	8.8	0	0	1.000	2	3.7	0	0	1.000	5	5.7	0	0	0.804
**International Working Group on Sarcopenia (IWGS)**															
**No sarcopenia**	34	100	5	100		52	100	8	100		86	100	13	100	
**Sarcopenia**	0	0	0	0		0	0	0	0		0	0	0	0	
*SMI for IWGS*															
**No sarcopenia**	32	100	5	100		52	100	7	100		84	100	12	100	
**Sarcopenia**	0	0	0	0		0	0	0	0		0	0	0	0	
*Gait speed for IWGS*															
**No sarcopenia**	17	56.7	5	100		31	59.6	8	100	0.067	48	58.5	13	100	0.010
**Sarcopenia**	13	43.3	0	0		21	40.4	0	0	0.067	34	41.5	0	0	0.010
**(Asian Working Group for Sarcopenia) AWGS**															
**No sarcopenia**	34	100	5	100		52	100	9	100		86	100	14	100	
**Sarcopenia**	0	0	0	0		0	0	0	0		0	0	0	0	
*SMI for AWGS*															
**No sarcopenia**	34	100	5	100		52	100	8	100		86	100	13	100	
**Sarcopenia**	0	0	0	0		0	0	0	0		0	0	0	0	
*Handgrip for AWGS*															
**No sarcopenia**	30	93.8	3	60	0.137	41	78.8	5	55.6	0.281	71	84.5	8	57.1	0.042
**Sarcopenia**	2	6.3	2	40	0.137	11	21.2	4	44.4	0.281	13	15.5	6	42.9	0.042
*Gait speed for AWGS*															
**No sarcopenia**	29	90.6	5	100	1.00	50	96.2	8	100	1.000	79	94	13	100	0.819
**Sarcopenia**	3	9.4	0	0	1.00	2	3.8	0	0	1.000	5	6	0	0	0.819
**FNIH**															
**No sarcopenia**	34	100	5	100		52	100	8	100		86	100	13	100	
**Sarcopenia**	0	0	0	0		0	0	0	0		0	0	0	0	
*ALM/BMI for FNIH*															
**No sarcopenia**	26	76.5	4	80	1.000	45	86.5	6	75	0.750	71	82.6	10	79.6	0.916
**Sarcopenia**	8	23.5	1	20	1.000	7	13.5	2	25	0.750	15	17.4	3	23.1	0.916
*Handgrip for FNIH*															
**No sarcopenia**	30	93.8	3	60	0.137	46	86.8	5	55.6	0.072	76	89.4	8	57.1	0.007
**Sarcopenia**	2	6.3	2	40	0.137	7	13.2	4	44.4	0.072	9	10.6	6	42.9	0.007
*gait speed for FNIH*															
**No sarcopenia**	29	90.6	5	100	1.000	51	96.2	9	100	1.000	80	94.1	14	100	0.785
**Sarcopenia**	3	9.4	0	0	1.000	2	3.8	0	0	1.000	5	5.9	0	0	0.785

**Table 3:** SARC-F Validated Against Different Sarcopenia Definitions.

	Sensitivity (%)			Specificity (%)		
	M	F	Total	M	F	Total
**Chair test**	33.3	23.1	25.9	100	100	100
**EWGSOP2 criteria**	/	/	/	87.2	86.9	87
*SMI*	/	/	/	87.2	85.5	86.1
*Handgrip*	20	36.4	31.3	88.2	90.4	89.5
*Gait speed*	0	0	0	86.1	85.2	85.6
**IWGS criteria**	/	/	/	87.2	86.7	86.9
*SMI*	/	/	/	86.5	88.1	87.5
*Gait speed*	0	0	0	77.3	79.5	78.7
**AWGS criteria**	/	/	/	87.2	85.2	86
*SMI*	/	/	/	87.2	86.7	86.9
*Handgrip*	50	26.7	31.6	90.9	89.1	89.9
*Gait speed*	0	0	0	85.3	86.2	85.9
**FNIH criteria**	/	/	/	87.2	86.7	86.9
*ALM*/*BMI*	11.1	22.2	16.7	86.7	88.2	87.7
*Handgrip*	50	36.4	40	90.9	90.2	90.5
*Gait speed*	0	0	0	85.3	85	85.1

## Data Availability

Not applicable.

## Supplementary Materials

The following supporting information can be downloaded at: https://www.mdpi.com/article/10.3390/nu14122533/s1, Supplementary File S1: SARC-F Italian version.
