# Supplementary material for: Validation of the Italian Version of the SARC-F Questionnaire to Assess Sarcopenia in Older Adults
Source: Nutrients. 2022 Jun 18;14(12):2533. doi: 10.3390/nu14122533 (PMC9228620; doi:10.3390/nu14122533)
Supplement: Supplementary file 1 [file nutrients-14-02533-s001.zip › nutrients-1755365-supplementary.pdf]

SARC-F Screening per la Sarcopenia VERSIONE ITALIANA

VARIABILI	DOMANDA	PUNTEGGIO
**FORZA**	Quanta difficoltà avverte nel sollevare e portare con sè un peso di circa 5 kg?	nessuno 0
		qualche 1
		molto (o non in grado) 2
**ASSISTENZA NELLA CAMMINATA**	Avverte difficoltà nel camminare all'interno di una stanza?	nessuno 0
		qualche 1
		molto (o non in grado) 2
**DIFFICOLTA' DI MOVIMENTO DA LETTO\SEDIA**	Quanta difficoltà avverte nel trasferirsi da una sedia al letto?	nessuno 0
		qualche 1
		molto (o non in grado) 2
**SALIRE LE SCALE**	Quanta difficoltà avverte a salire una rampa di 10 scale?	nessuno 0
		qualche 1
		molto (o non in grado) 2
**CADUTE**	Quante volte è caduto a terra durante l'ultimo anno	nessuno 0
		qualche 1
		molto (o non in grado) 2

Punteggio totale________________
